# NUMB regulates the endocytosis and activity of the anaplastic lymphoma kinase in an isoform-specific manner

**DOI:** 10.1093/jmcb/mjz003

**Published:** 2019-02-06

**Authors:** Ran Wei, Xuguang Liu, Courtney Voss, Wentao Qin, Lina Dagnino, Lei Li, Marc Vigny, Shawn Shun-Cheng Li

**Affiliations:** 1 Departments of Biochemistry, Schulich School of Medicine and Dentistry, Western University, London, Ontario, Canada; 2 Dentistry, Schulich School of Medicine and Dentistry, Western University, London, Ontario, Canada; 3 Physiology and Pharmacology and Schulich School of Medicine and Dentistry, Western University, London, Ontario, Canada; 4 School of Basic Medical Sciences, Qingdao University, Qingdao, Shandong, China; 5 Université Pierre et Marie Curie, UPMC, INSERM UMRS-839, Paris, France; 6 Oncology, Schulich School of Medicine and Dentistry, Western University, London, Ontario, Canada

**Keywords:** ALK, NUMB, isoform, endocytosis, degradation, recycling

## Abstract

NUMB is an evolutionarily conserved protein that plays an important role in cell adhesion, migration, polarity, and cell fate determination. It has also been shown to play a role in the pathogenesis of certain cancers, although it remains controversial whether NUMB functions as an oncoprotein or tumor suppressor. Here, we show that NUMB binds to anaplastic lymphoma kinase (ALK), a receptor tyrosine kinase aberrantly activated in several forms of cancer, and this interaction regulates the endocytosis and activity of ALK. Intriguingly, the function of the NUMB–ALK interaction is isoform-dependent. While both p66-NUMB and p72-NUMB isoforms are capable of mediating the endocytosis of ALK, the former directs ALK to the lysosomal degradation pathway, thus decreasing the overall ALK level and the downstream MAP kinase signal. In contrast, the p72-NUMB isoform promotes ALK recycling back to the plasma membrane, thereby maintaining the kinase in its active state. Our work sheds light on the controversial role of different isoforms of NUMB in tumorigenesis and provides mechanistic insight into ALK regulation.

## Introduction

Anaplastic lymphoma kinase (ALK) is a member of the orphan receptor tyrosine kinase (RTK) family. It was originally described as a fusion protein of the t(2;5) (p23;q35) translocation expressed in anaplastic large cell lymphomas (ALCL) (Morris et al., 1994; Shiota et al., 1994). Normally, the ALK receptor is only transiently expressed in the nervous system during early developmental stages and maintained at a low level in adult tissue except for some neuronal cells (Morris et al., 1994). This gene is considered non-essential for development as only a mild behavioral phenotype was observed in knockout mice (Bilsland et al., 2008). However, deregulation of ALK activity—including mutations, amplifications, and translocations—has been characterized in a subset of cancers. For instance, two ALK constitutively-active mutants, F1174L and R1275Q, were frequently found in neuroblastomas, which accounts for 15% of all childhood cancer deaths (George et al., 2008). ALK amplification has been detected in neuroblastoma, colorectal cancer and non-small cell lung cancer (NSCLC) (Salido et al., 2011; Bavi et al., 2013; Wang et al., 2013). It should be noted that 4%–7% of NSCLC involves an EML4-ALK fusion kinase that is constitutively activated and shown to promote and maintain the malignant behavior of the cancer cells (Soda et al., 2007; Sasaki et al., 2010). The aberrantly activated ALK promotes tumorigenesis by activating various cancer-related signaling pathways, including the MAP kinase proliferation and PI3K survival pathways (Mosse et al., 2009). Since ALK levels are low in healthy adult tissues, selective ALK inhibition tends to result in less adverse effects without compromising efficacy (Crescenzo and Inghirami, 2015). The FDA granted accelerated approvals in 2011 and 2014 for two ALK inhibitors, namely crizotinib and ceritinib, in treating ALK positive metastatic NSCLC. Additionally, the inhibitor alectinib was recently approved for NSCLC patients who could not tolerate critizonib.

Despite the oncogenic potential and therapeutic value of ALK, the mechanism of ALK regulation is not fully understood. Here, we show that NUMB, the endocytic adaptor protein interacts, binds to ALK and regulates the intracellular trafficking and degradation of the latter. Curiously, we have found that two NUMB isoforms, p72 and p66, play distinct roles in regulating ALK endocytosis and activity. NUMB is a membrane-associated protein that is highly conserved from fly to human. It plays an essential role in the maintenance of cellular homeostasis in the nervous system (Dho et al., 1999; Pece et al., 2011). The *numb* gene was the first isolated cell fate determinant from *Drosophila*, whereby asymmetric distribution of NUMB during mitosis produced diverse neural cell fates (Uemura et al., 1989). In tumorigenesis, NUMB functions as an adaptor, and its asymmetric distribution has been implicated in a variety of cancer-related events and signaling pathways (Gulino et al., 2010; Pece et al., 2011). NUMB is generally considered a tumor suppressor. This is due to its negative regulation of the Notch signaling pathway and cell proliferation by promoting Notch endocytic degradation (McGill and McGlade, 2003; McGill et al., 2009; Couturier et al., 2012), or by preventing MDM2-mediated ubiquitination of p53 (Colaluca et al., 2008). Nevertheless, NUMB has also been shown to promote tumorigenesis in an isoform-specific manner (Bechara et al., 2013).

The *numb* transcript can undergo alternative splicing to produce isoforms. The four isoforms well characterized in both mice and human beings share a similar structure and contain an N-terminal phosphotyrosine binding (PTB) domain, a proline rich region (PRR), two Asp–Pro–Phe (DPFs) motifs, and one Asn–Pro–Phe (NPF) motif at the C-terminal. The isoforms differ in the inclusion or exclusion of exon 3 in the PTB domain or exon 9 in the PRR region (Bork and Margolis, 1995; Verdi et al., 1996, 1999; Salcini et al., 1997; Dho et al., 1999). The four NUMB isoforms have been shown to exhibit distinct functions in *Drosophila* neuronal development (Verdi et al., 1999), with the two short-PRR isoforms primarily stimulating neuronal differentiation, while the long-PRR isoforms promoting proliferation. Similarly, the long-PRR isoforms have been associated with multiple cancer types, including breast cancer, colon cancer and lung cancer (Misquitta-Ali et al., 2011). It has also been reported that the RBM5/6 and RBM10 splicing factors, which are responsible for splicing exon 9 in the *numb* transcript, play a role in cancer cell proliferation (Bechara et al., 2013). While the molecular basis underlying the different functions of the various NUMB isoforms is not yet clear, it is speculated that the exon 9-inclusion NUMB isoforms p72 and p71 may act antagonistically to the exon 9-skipping isoforms p66 and p65 in cell proliferation. Indeed, in some lung cancer cells, it has been shown that inclusion of exon 9 is correlated with increased cell proliferation (Westhoff et al., 2009; Bechara et al., 2013).

We show here that the p65/p66-NUMB and p71/p72-NUMB isoforms play distinct roles in tumorigenesis through ALK. We identified ALK as a NUMB-binding protein from a high throughput screen (Wei et al., 2018). We found that the two proteins bind directly to each other through the NUMB PTB domain and the N^1477^MAF^1480^ and N^1585^YGY^1586^ motifs in ALK. Moreover, we found that both NUMB isoforms could promote ALK endocytosis. Intriguingly, in post-endocytic trafficking, the p66-NUMB isoform promoted ALK degradation via the lysosomal pathway, thereby decreasing the activity of ALK and the downstream MAP kinase proliferation signal. Conversely, the p72-NUMB isoform facilitated the recycling of ALK back to the plasma membrane, thereby maintaining ALK activity in cells. Our data provide mechanistic insight into ALK regulation and the tumorigenic potential of the p72-NUMB isoform, with potential clinical implications.

## Results

### Identification of ALK as a novel NUMB-binding protein

**Figure 1:**
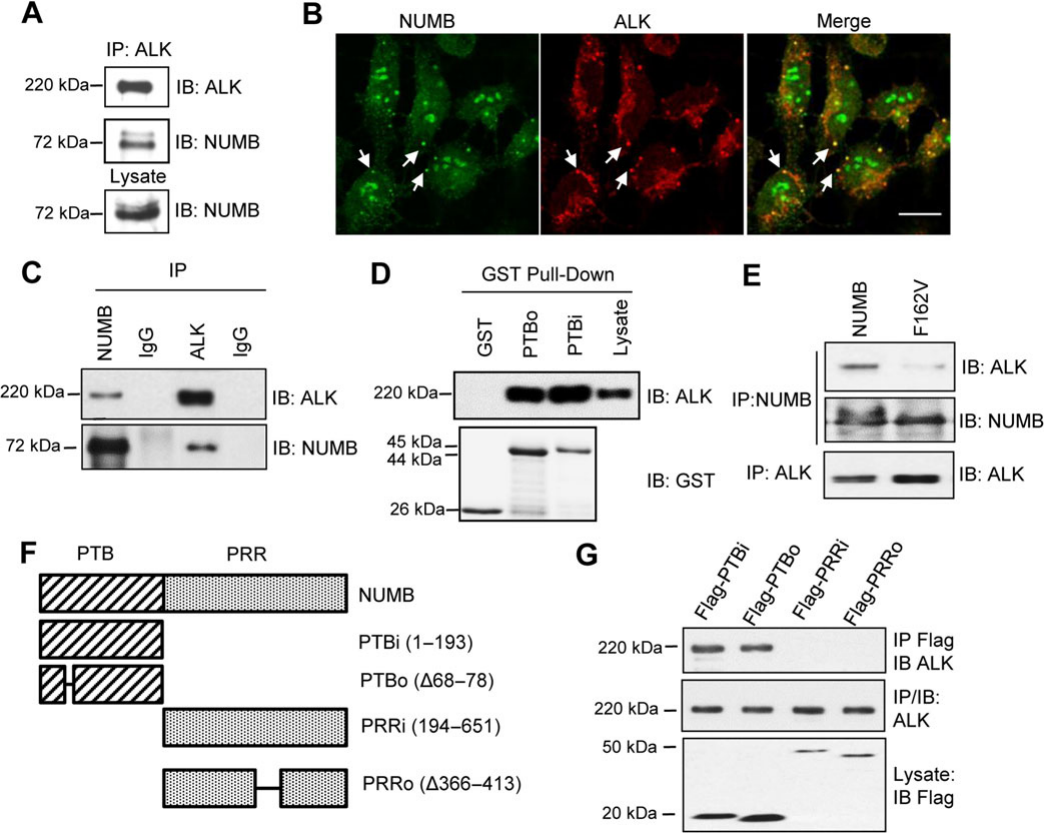
NUMB interacts with ALK. (**A**) Endogenous NUMB and ALK co-immunoprecipitate (IP) from IMR-5 neuroblastoma cells. IB, immunoblot. (**B**) NUMB and ALK co-localized in IMR-5 cells as shown by the confocal immunofluorescence of NUMB (green) and ALK (red). Co-localization was found in the cell membrane and dot formations (arrows). Scale bar, 10 μm. (**C**) Endogenous NUMB interaction with ectopically expressed ALK in the HEK293/ALK stable cell line. (**D**) The NUMB PTB domain bound directly to ALK. PTBi/PTBo, PTB domains with/without an 11-aa insert. (**E**) The NUMB–ALK interaction was greatly attenuated for the PTB-deficient mutant F162V. (**F** and **G**) NUMB truncation mutants (Flag-tagged) were used for binding studies by expression in the HEK293/ALK cells.

The PTB domain, which is capable of binding to a variety of different proteins, plays a critical role in NUMB function (Gulino et al., 2010). Previously, we isolated several NUMB PTB-binding receptor tyrosine kinases, including ALK, from a high throughput screening for PTB binding partners (Wei et al., 2018). We verified the endogenous NUMB–ALK interaction by co-immunoprecipitation (Co-IP) in the IMR-5 cells (Figure 1A), a neuroblastoma cell line expressing wild-type ALK (George et al., 2008; Tumilowicz et al., 1970). In addition, by co-immunostaining NUMB and ALK, we found that they partially co-localized with each other in IMR-5 cells. The co-localization signals were presented in punctate patterns and were mainly located in the peripheral region of the plasma membrane (Figure 1B). This suggests that the NUMB–ALK interaction may occur in endocytic vesicles.

To facilitate functional studies, we created HEK293 cells transiently or stably expressing ALK and/or NUMB. Using the HEK293/ALK cell line stably expressing wild-type (wt) ALK (Moog-Lutz et al., 2005), we validated the NUMB–ALK interaction by Co-IP (Figure 1C). We also used GST-fused PTB domains with or without exon 3, which encodes an 11-aa sequence insert, i.e. GST-PTBi with the insert and GST-PTBo without the insert (Dhami et al., 2013), to pull down ALK from the same cell line. Both GST fusion proteins, but not GST itself, were capable of binding to ALK (Figure 1D). To investigate whether the PTB domain was sufficient for ALK binding, we created a NUMB mutant bearing the F162V mutation within the PTB domain that was found previously to render the PTB domain incapable of ligand-binding (Zwahlen et al., 2000). Upon its expression in the HEK293/ALK cells, a marked reduction of binding to ALK was seen for the F162V mutant compared to wt NUMB (Figure 1E), indicating that the PTB domain played a critical role in the NUMB–ALK interaction. Moreover, using a series of NUMB mutants in which the PTB or PRR was deleted (Figure 1F), we confirmed that the PTB domain, regardless of the presence (PTBi) or absence of the exon 3 insert (PTBo), but not the PRR, was responsible for ALK binding (Figure 1G).

**Figure 2:**
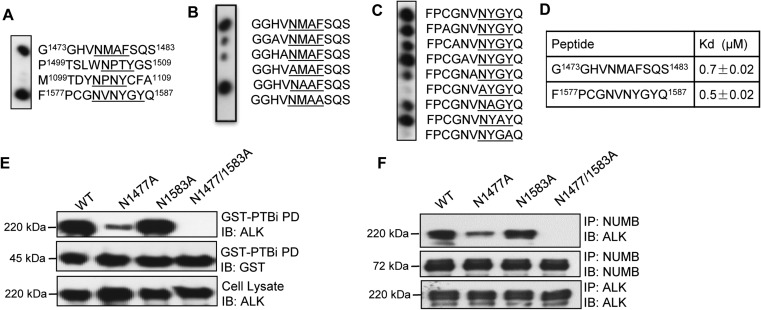
NUMB binds to ALK via the NxxF/Y motif. (**A**) Binding of the PTBi domain (in the GST-PTBi form) to four ALK-derived peptides containing the NxxF/Y motif (where x represents any amino acid). (**B** and **C**) Binding of the PTBi domain to Ala-scanning arrays of the G^1473^GHVNMAFSQS^1483^ (**B**) and F^1577^PCGNVNYGYQ^1587^ (**C**) peptides. Anti-GST Far-Western was used to detect the bound protein. (**D**) Affinities (Kd) of the two peptides for the GST-PTBi in solution, determined by fluorescence polarization. (**E** and **F**) The N1477A single mutant and N1447/N1583 double mutant were defective in binding to PTBi in a GST-pulldown assay (**E**) and the full-length NUMB in Co-IP (**F**). PD, pulldown.

The NUMB PTB domain is known to bind to NxxF/Y motifs (Li et al., 1998). We found that the intracellular region of ALK contains four such motifs. The corresponding peptides, G^1473^GHVNMAFSQS^1483^, P^1499^TSLWNPTYGS^1509^, M^1099^TDYNPNYCFA^1109^, and F^1577^PCGNVNYGYQ^1587^, were synthesized on a cellulose membrane and blotted with GST-PTBi in a Far-Western assay. Only G^1473^GHVNMAFSQS^1483^ and F^1577^PCGNVNYGYQ^1587^ were found to bind the PTB domain (Figure 2A). These two peptides were then subjected to alanine-scanning substitution and the resulting peptide arrays blotted with GST-PTBi as well Figure 2B and C). The binding data further confirmed the importance of the N^1477^MAF and N^1583^YGY motifs in PTB binding and the critical role for the N and F/Y residues within the motifs (Wang et al., 2009). Next, the binding affinities between the two peptides and the PTBi domain were determined by fluorescence polarization (FP) assay. The dissociation constants (K_D_) between fluorescein-labeled G^1473^GHVNMAFSQS^1483^ or F^1577^PCGNVNYGYQ^1587^ peptide and PTBi were 0.7 μM and 0.5 μM, respectively (Figure 2D). To verify these binding interactions in cells, we created ALK mutants in which either or both of Asn^1477^ and Asn^1583^ were mutated to Ala in the N1477A and N1583A mutants. The resultant mutants were expressed in HEK293 cells, and the cell lysates were incubated with GST-NUMB-PTBi. As shown in Figure 2E, the ALK single mutant N1477A displayed significantly weakened NUMB binding, while the N1477/N1583 double mutant completely lost the interaction. Identical results were obtained from Co-IP of NUMB with ALK or the mutants (Figure 2F). Collectively, these data demonstrate that the PTB domain of NUMB and the N^1477^MAF and N^1577^YGY motifs of ALK mediate the binding of the corresponding proteins via direct physical interaction.

### NUMB regulates ALK endocytosis

**Figure 3:**
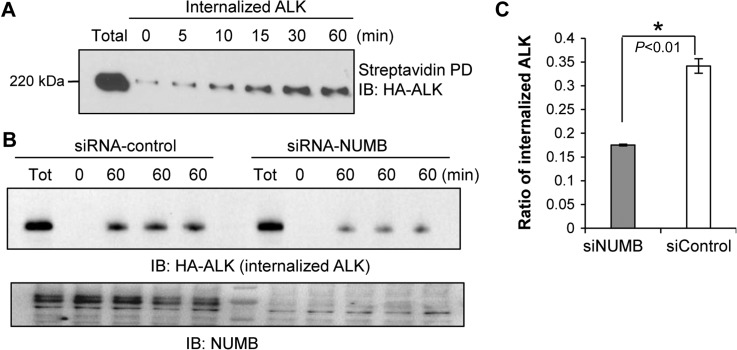
NUMB promotes ALK internalization. (**A**) ALK was internalized from the plasma membrane to the cytosol. The internalization of ALK was determined by biotinylation internalization assay. (**B** and **C**) siRNA-mediated depletion of NUMB from the HEK293/ALK cells significantly attenuated ALK internalization. A representative western blot for the internalized ALK (HA-ALK) is shown in **B** and the quantitation is shown in **C**. *P < 0.01, *n* = 3.

Inasmuch as NUMB has been shown to be involved in the endocytosis of a number of membrane proteins (Gulino et al., 2010), we hypothesized that NUMB may play a similar role in regulating ALK endocytosis. To validate this hypothesis, we examined ALK endocytosis by cell-surface biotinylation and internalization assay. Specifically, in HEK293/ALK cells, membrane-localized HA-ALK was labeled with NHS-SS-biotin and then the cells were cultured for up to 60 min under normal conditions to allow internalization to occur. The internalized biotinylated HA-ALK was pulled down by streptavidin beads at various time points. The samples were then probed with HA antibody to determine the relative amount of internalized ALK in the cytoplasm. We observed that ALK internalization occurred very rapidly and appeared to plateau at approximately 30 min (Figure 3A). To further investigate the possible function of NUMB in this process, we depleted the NUMB protein level by RNA interference in the HEK293/ALK cells. This was followed by cell-surface biotinylation and internalization assays. The NUMB knockdown cells exhibited significantly less internalized biotinylated ALK in the cytoplasm at the same time points (e.g. 60 min) compared to that in cells transfected with the scrambled control siRNA (Figure 3B). Quantitatively, approximately 18% of surface labeled ALK was internalized after 60 min in NUMB knockdown cells, while this number was around 34% in control cells (Figure 3C).

### NUMB regulates post-endocytic trafficking of ALK in an isoform-dependent manner

The function of NUMB in endocytosis was previously investigated by studying NUMB-regulated NOTCH receptor internalization (McGill et al., 2009). The NUMB p66 isoform was found to promote Notch endocytosis and degradation, which, in turn, inhibited NOTCH-mediated cell proliferation. However, alternatively spliced NUMB could also increase the activity of NOCTH and its downstream signaling (Westhoff et al., 2009; Bechara et al., 2013). It has also been shown that different NUMB isoforms play distinct roles tumorigenesis (Misquitta-Ali et al., 2011). Mammalian NUMB has four isoforms resulting from alternative splicing. The p72 isoform contains both exon 3 and exon 9, the p71 isoform lacks exon 3, the p66 isoform lack exon 9, and the p65 isoform contains neither exon. Since both PTBi and PTBo bind to ALK (Figure 1A, B, and G), we speculate that p72/p71 may play a different role in post-endocytic trafficking of the ALK receptor compared to p66/p65. Instead of promoting receptor degradation, p72/p71 may cause the recycling of the receptor back to the cell membrane to promote the activity of the receptor, which is also a default post-endocytic destination of internalized membrane receptors (Goh and Sorkin, 2013). Based on a previous study on the subcellular localization of NUMB isoforms, p72 and p66 tend to localize to the periphery of the cell membrane, while p71 and p65 typically localize in the cytoplasm (Dho et al., 1999). Thus, in the following endocytosis study, we focused on the isoforms that generally interact with membrane localized receptors: p72 and p66.

**Figure 4:**
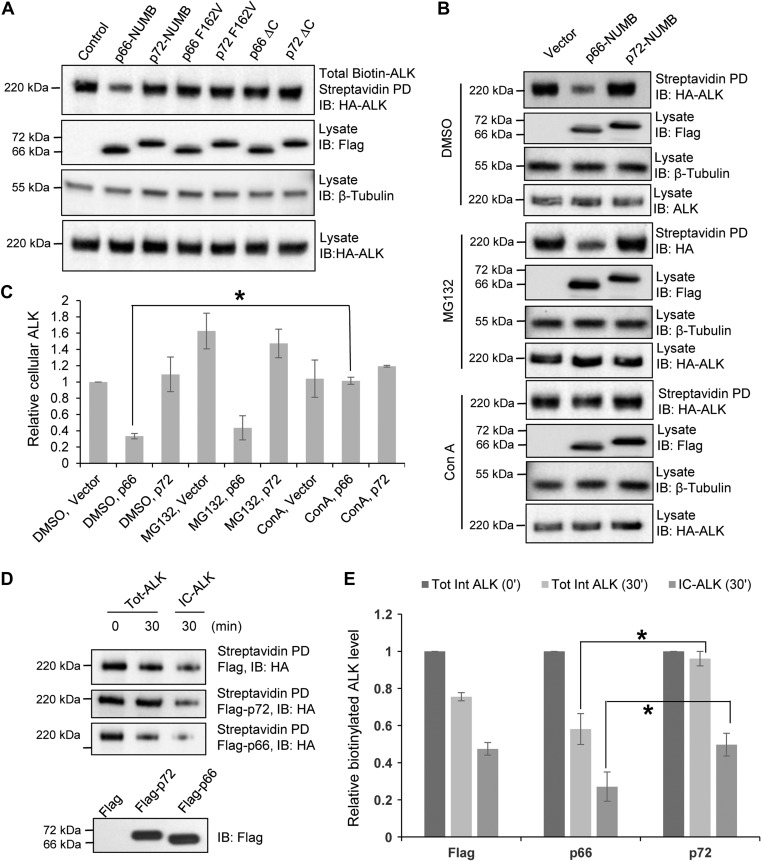
NUMB isoforms play distinct roles in ALK post-endocytic trafficking. (**A**) p66-NUMB, but not p72-NUMB, promoted post-endocytic ALK degradation. HEK293/ALK cells were transfected with the indicated NUMB isoforms and mutants. The p66 F162V and p72 F162V are NUMB mutants with a disabled PTB domain (Zwahlen et al., 2000). p66 ΔC and p72 ΔC are NUMB mutants shown to be deficient in endocytosis (McGill et al., 2009). The total amount of cell surface ALK is determined by cell surface protein biotinylation and streptavidin pulldown assay followed by western blotting. (**B** and **C**) The p66-NUMB isoform promoted degradation of endocytosed ALK via the lysosomal pathway. HEK293/ALK cells were transfected with the empty vector or p66-NUMB or p72-NUMB (Flag-tagged) expression constructs. The transfected cells were treated with the vehicle DMSO, the proteasome inhibitor MG132 (10 μM), or the lysosome inhibitor Concanamycin A (Con A, 100 nM). ALK remaining on the plasma membrane was determined by cell surface protein biotinylation and streptavidin pulldown assay. Flag and HA IBs were used to show Numb and ALK protein expression whereas the β-tubulin IB was included for loading control. *P < 0.05, *n* = 3. (**D** and **E**) p72-NUMB but not p66-NUMB isoform promoted post-endocytic ALK recycling. HEK293/ALK cells were transfected with expression construct for Flag-p66-NUMB or Flag-p72-NUMB or the empty vector. Shown are western blots of total or intracellular (IC) ALK at 0 or 30 min of the ALK recycling assay. *P < 0.05, *n* = 3.

The p72 and p66 isoforms were transiently expressed with a Flag-tag in the HEK-293/HA-ALK cells. Cell-surface biotinylation assay was carried out 48 h after transfection. Next, the total biotinylated HA-ALK protein was pulled down with streptavidin beads 1 h after biotinylation and the samples were probed with an anti-HA antibody. The p66 isoform was found to significantly decrease the total biotinylated ALK level whereas the p72 isoform exhibited little effect compared to the vector control. This suggests that p66 promoted post-endocytic ALK degradation. In addition, the PTB deficient p66-F162V mutant and a mutant p66 lacking the C-terminal endocytosis motif exhibited no apparent effect on the biotinylated ALK level, implicating both the PTB domain and the endocytic motif in the function of p66 (Figure 4A). Remarkably, the equivalent p72 mutants had no effect on the biotinylated ALK level, indicating that p72-NUMB is not involved in ALK degradation.

The decrease in biotinylated cell surface ALK caused by p66 could be due to proteasome or lysosome mediated degradation. To identify the degradation pathway, we treated cells expressing p72 or p66 isoform with the lysosome inhibitor concanamycin A (ConA) or the proteasome inhibitor MG132 prior to the endocytosis assay. Compared to the vehicle DMSO, MG132 treatment did not make a significant difference in the biotinylated ALK in all treated cells regardless of whether they expressed the p72 or p66 isoforms of NUMB. In contrast, ConA treatment led to a specific increase in biotinylated ALK in cells expressing the p66 isoform (Figure 4B and C). These data indicate that the p66, but not the p72 isoform of NUMB, promote the degradation of endocytic ALK via the lysosome pathway.

To further characterize the function of NUMB in ALK recycling, we carried out biotinylation and recycling assays in HEK293/ALK cells expressing the p72 or p66 isoform. The cells were first surface-biotinylated and incubated at 18°C for 2 h to allow internalization to occur, but not recycling of biotinylated ALK. Next, the cells were treated with glutathione (GSH) to remove biotin from the cell surface. Following surface biotin removal, the cells were either lysed for examining the total amount of internalized/biotinylated ALK (Tot-ALK at 0 min) or returned to 37°C to resume normal endocytic trafficking (endocytosis/recycling/degradation). After 30 min of incubation at 37°C, half of the cells were directly lysed (Tot-ALK at 30 min), while the other half were lysed after GSH treatment that removed any biotin from protein recycled back to the cell surface (IC-ALK at 30 min). The biotinylated ALK was pulled down using streptavidin beads and blotted with HA antibody. As shown in Figure 4D, the Tot-ALK level was significantly decreased in cells expressing the p66 isoform after 30 min of endocytosis recovery at 37°C. This was consistent with the results presented in Figure 4A. However, only a minor change was observed in cells expressing the p72 isoform. A relative quantification of western blotting better illustrates the difference between the isoforms (Figure 4E). After 30 min without the second GSH treatment, the biotinylated ALK level was ~96% of the initial level when p72 isoform was expressed, while only ~58% of that when p66 isoform was expressed. Upon the second GSH treatment, the biotinylated ALK levels were ~50% and ~27% in cells expressing p72 and p66, respectively. This implies that p72 expression resulted in ~46% (96%–50%) of ALK recycling and p66 expression resulted in ~31% (58%–27%) of ALK recycling. These data indicate that the p66 and p72 isoforms play significantly different roles in the intracellular trafficking and degradation of ALK.

**Figure 5:**
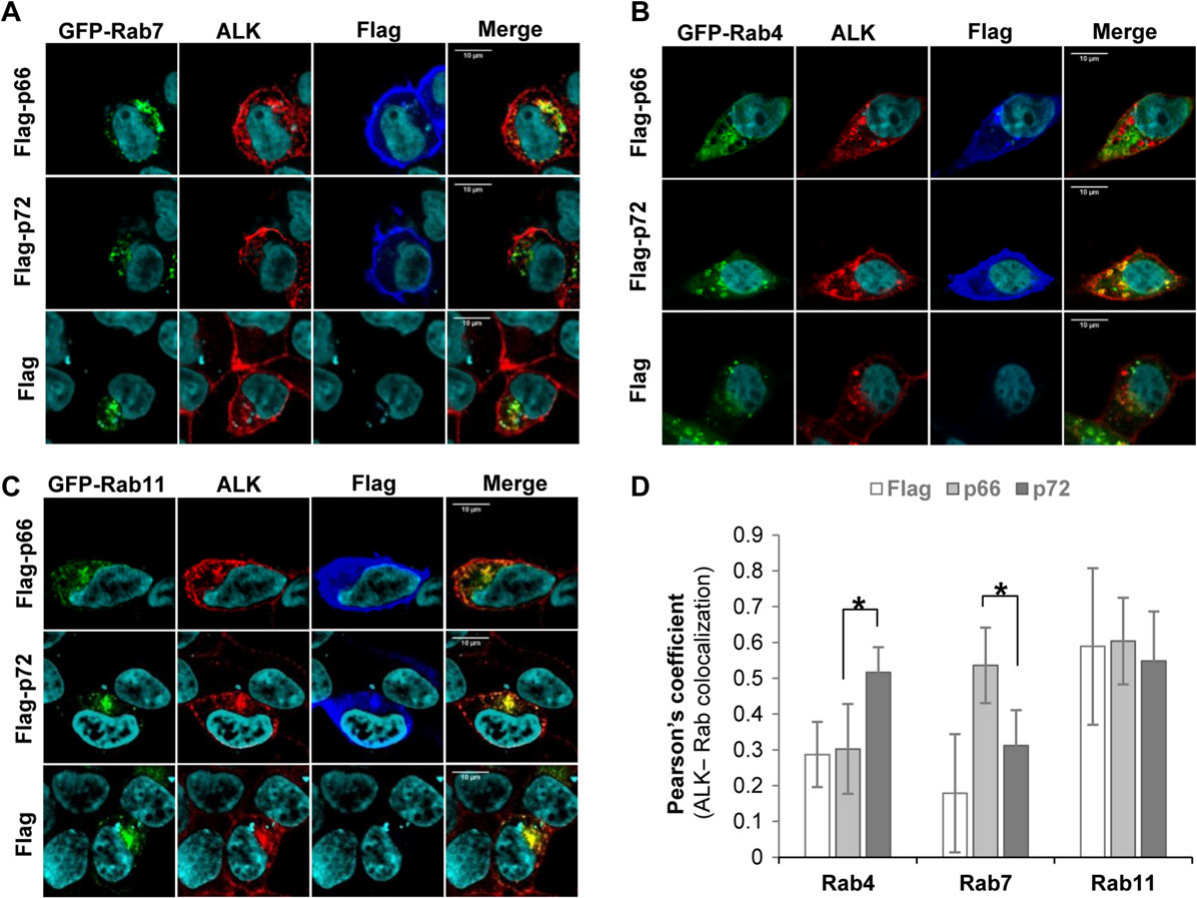
NUMB isoforms differentially co-localize with Rab4 and Rab7. HEK293/ALK cells were co-transfected with plasmids for Flag-labeled p66-NUMB or p72-NUMB and GFP-fused Rab4, Rab7, or Rab11. Co-immunostaining was then carried out using mAb-46 (against ALK, red, 1:1000) and anti-Flag (for Flag-NUMB, blue, 1:1000). (**A**) p66-NUMB expression promoted the co-localization of ALK and the late endosome marker Rab7. (**B**) p72-NUMB expression promoted co-localization between ALK and the early endosome marker Rab4. (**C**) Another early endosome marker, Rab11, generally co-localized with ALK in all conditions. (**D**) Quantification of the co-localization data in **A**–**C** based on the corresponding Pearson’s co-efficient. NUMB isoforms-transfected cells (p66 vs. p72): Rab4, *P < 0.01, *n* = 8; Rab7, *P < 0.01, *n* = 5; Rab11, *n* = 5. Flag empty vector-transfected cells: Rab4, *n* = 6; Rab7, *n* = 8; Rab11, *n* = 9. Scale bar in **A**–**C**, 10 μm.

To understand the mechanism underpinning the different functions of the two NUMB isoforms in ALK degradation and recycling, we examined their co-localizations with Rab4, Rab7, and Rab11, which were expressed as GFP-fused proteins in HEK293/ALK cells together with Flag-p66-NUMB or Flag-p72-NUMB. There was a significant increase in co-localization between ALK and the late endosome marker GFP-Rab7 (Chavrier et al., 1990) in HEK293/ALK cells co-expressing the p66, but not the p72 isoform of NUMB or the vector control (Figure 5A). This suggests that p66-NUMB may facilitate the degradation of ALK by routing it to the late endosome and lysosome. In contrast, expression of p72-NUMB, but not p66-NUMB, promoted the co-localization of ALK with the early endosome marker Rab4 (Van Der Sluijs et al., 1991) (Figure 5B). Because the recycling process is dependent on both Rab4 and Rab11 and/or Rab14 (Pfeffer, 2013), we also examined the co-localization of ALK with Rab11 in cells expressing either NUMB isoform. Intriguingly, neither NUMB isoform could significantly promote the co-localization of ALK and Rab11 (Figure 5C). These observations were further verified by a quantitative analysis of Pearson’s co-efficient from multiple cells/images (Figure 5D), which showed significant differences in ALK colocalization with Rab4 or Rab7 in cells expressing the p66 or p72 isoform.

Collectively, the above data demonstrate that NUMB plays an important role in the sorting of endocytosed ALK. Specifically, p72-NUMB facilitates the fast-recycling of ALK via Rab4-containing early endosomes, while p66-NUMB promotes the lysosome-dependent degradation of ALK via the Rab7-containing late endosomes.

### NUMB isoforms play distinct roles on ALK activity by differential regulation of ALK trafficking and degradation

**Figure 6:**
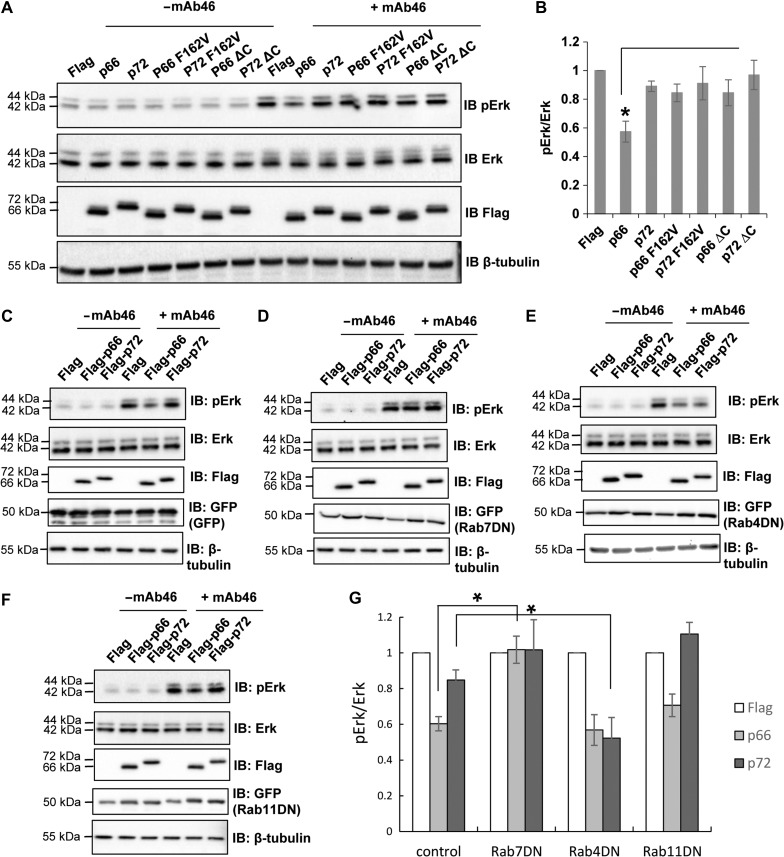
p66-NUMB inhibits ALK-mediated MAPK signaling. (**A** and **B**) Erk phosphorylation was attenuated by p66-NUMB expression in HER293/ALK cells. ALK was activated by treating the cells with the anti-ALK antibody mAb46. (**B**) Quantification of the pErk/Erk ratio based on IBs in **A**. The differences between cells expressing p66-NUMB and cells expressing p72-NUMB or NUMB mutant p66 F162V, p72 F162V, p66 ΔC, or p72 ΔC are significant. *P < 0.01, *n* = 3. (**C**–**F**) Interrupting endocytosis with dominant-negative mutants of Rab7, Rab4, and Rab11 led to differential effects on ALK regulation by NUMB. (**D**) Overexpression of the Rab7DN mutant eliminated the inhibitory effect of p66-NUMB on ALK activity (Erk phosphorylation). (**E**) Overexpression of the Rab4DN mutant prevented p72-NUMB from maintaining ALK activity by recycling. (**F**) Overexpression of Rab11DN mutant had little effect. (**G**) Quantification of data from **C**–**F** to show changes in pErk/Erk ratio under different conditions. *P < 0.01, *n* = 3.

ALK signals through the MAP kinase pathway to regulate cell proliferation (Hallberg and Palmer, 2013). To interrogate the role of the two NUMB isoforms in ALK activity, we examined the Erk phosphorylation in HEK293/ALK cells expressing either wildtype or ALK-binding deficient p66/p72-NUMB. We used an antibody for the extracellular region of ALK, mAb46, to specifically stimulate the activation of ALK and MAP kinase pathway (Erk phosphorylation) under serum-free conditions (Moog-Lutz et al., 2005). As shown in Figure 6A, Erk phosphorylation was promoted by the mAb46 treatment in HEK293/ALK cells. Co-expression of p66-NUMB significantly attenuated Erk phosphorylation, while the ALK-binding deficient mutant p66 F162V or the endocytosis deficient form p66 ΔC had little effects (Figure 6A and B).

To further validate that the isoform-specific function of NUMB on ALK activation is dependent on their roles in ALK post-endocytic trafficking, we used the dominant-negative forms of Rab4, Rab7, or Rab11 (GFP-Rab4/7/11DN) to specifically perturb endocytosis routes in HEK293/ALK co-expressing Flag-p66-NUMB, Flag-p72-NUMB, or Flag only. Since Rab7 plays a critical role in controlling late endocytic structures/lysosome (Bucci et al., 2000), we predicted that the GFP-Rab7DN mutant would prevent the internalized ALK from lysosomal degradation. Indeed, we observed that the expression of GFP-Rab7DN eliminated a decrease in the Erk phosphorylation levels mediated by p66-NUMB (Figure 6C, D, and G). These results suggested that the inhibitory effect of p66-NUMB on ALK activation is dependent on its ability to sort ALK to the lysosome for degradation (Figure 4B and C). Blocking this process with the GFP-Rab7DN would lead to retention of the internalized ALK in the late endosome, thereby enhancing Erk activation. Presumably, ALK was still able to signal through Erk at the endosomal membrane as has been shown for a number of membrane receptors, including receptor tyrosine kinases (Murphy et al., 2009). In complementary experiments, we observed that blocking the fast recycling endosomes by the GFP-Rab4DN (Yudowski et al., 2009) led to a ~40% reduction in the Erk phosphorylation in p72-NUMB expressing cells. However, it had little effect in the p66-NUMB expressing cells (Figure 6C, E, and G). In contrast, blocking slow recycling of endocytosed receptor by GFP-Rab11DN had no significant effect on the Erk activation by ALK in cells co-expressing either p66-NUMB or p72-NUMB (Figure 6C, F, and H). Collectively, these data corroborate with our earlier observation that p72-NUMB facilitates ALK recycling back to the plasma membrane whereas p66 promotes ALK degradation in the lysosome.

**Figure 7:**
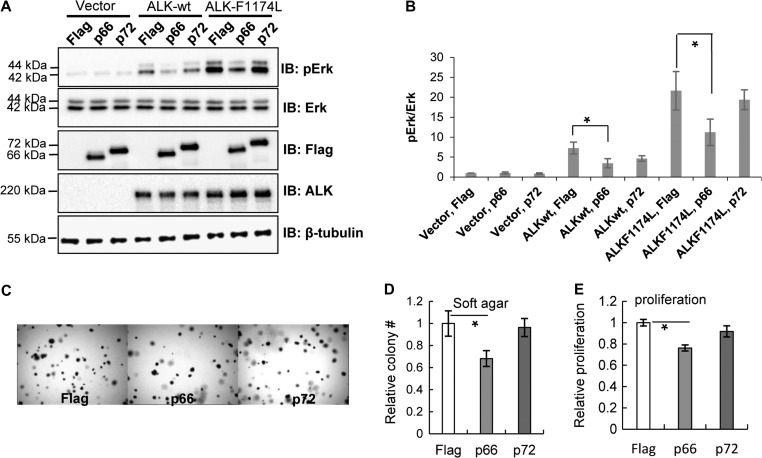
p66-NUMB inhibits ALK-dependent cell growth in culture medium and soft agar. (**A**) p66-NUMB significantly inhibited the Erk activation (pErk) in HEK293 cells expressing wild-type ALK or the oncogenic mutant F1174L. (**B**) Quantification of immunoblots in **A**. *P < 0.05, *n* = 3. (**C** and **D**) p66-NUMB significantly inhibited anchorage-independent cell growth of HEK293 cells expressing the ALK-F1174L mutant. *P < 0.01, *n* = 3. (**E**) p66-NUMB significantly inhibited the proliferation of HEK293-ALK-F1174L cells. WST-8 assay was carried out 48 h after transfection. *P < 0.01, *n* = 3.

In certain tumors, ALK can be activated by gene fusion or mutation. A recent study has identified several missense mutations in ALK associated with cancer (Chen et al., 2008; George et al., 2008; Janoueix-Lerosey et al., 2008; Mosse et al., 2008). We thus evaluated the activity of one such mutant, ALK-F1174L, in the presence of p66-NUMB or p72-NUMB in HEK293 cells. As shown in Figure 7A, while both wt and mutant ALK stimulated the activation of MAP kinase pathway under the normal cell culturing conditions with 10% serum, the F1174L mutant was associated with a significantly higher Erk phosphorylation level compared to the wt ALK. Curiously, this higher Erk phosphorylation was greatly attenuated by co-expression p66-NUMB, but not p72-NUMB (Figure 7A and B). To determine the effect of p66 vs. p72 on the tumorigenicity of the ALK-F1174L mutant, we carried out cell proliferation assays on soft-agar or culture medium for the HEK293-ALK-F1174L cells co-expressing p66-NUMB or p72-NUMB. We found that, compared to cells transfected with the empty Flag vector, the p66-NUMB, but not p72-NUMB, significantly inhibited colony-formation in soft-agar (Figure 7C and D). Similarly, p66-NUMB, but not p72-NUMB significantly inhibited cell growth in liquid culture (Figure 7E). Therefore, p66-NUMB and p72-NUMB act antagonistically in regulating ALK and MAP kinase activities via routing ALK to different endocytic pathways.

## Discussion

The NUMB protein was the first described cell fate determinant which is conserved from fly to human (Uemura et al., 1989; Verdi et al., 1999, 1996). Further studies have since revealed the complex nature of NUMB functions in a wide range of physiological or pathological processes. In tumorigenesis, NUMB is defined as a tumor suppressor for its role in binding and regulating the NOTCH receptor and p53 (McGill and McGlade, 2003; Colaluca et al., 2008; McGill et al., 2009; Couturier et al., 2012). Interestingly, the NUMB–NOTCH and NUMB–p53 interactions take place in the cell membrane and nucleus, respectively, and suppress tumorigenesis via distinct mechanisms, underscoring the multifaceted roles of NUMB. The function of NUMB is critically dependent on the interaction between its PTB domain and the binding partner that frequently contain an NxxF/Y motif (Li et al., 1998; Zwahlen et al., 2000). Besides the PTB domain, NUMB also contains a proline-rich region (PRR) and binding motifs for endocytic regulators such as EH, EPS15, EPS15R, and AP-2 (Gulino et al., 2010). Whereas the PTBi and PTBo isoforms exhibit little difference in binding specificity (Dho et al., 1999; Dhami et al., 2013; Wang et al., 2018), the PRRi and PRRo isoforms have distinct functions in cell proliferation and differentiation during development (Dho et al., 1999; Verdi et al., 1999; Dooley et al., 2003; Yoshida et al., 2003; Bani-Yaghoub et al., 2007). While it remains to be rigorously tested, it is speculated that the NUMB PTB domain may mainly serve as a protein recognition module while the non-PTB regions are responsible for recruiting other effectors, including endocytic proteins. Consistently, our work demonstrates that both the PTBi and PTBo isoforms bind to ALK through the NxxF/Y motifs. However, the PRRi-containing NUMB isoform, p72, mediates ALK recycling, whereas the PRRo-containing isoform, p66, mainly promotes ALK degradation. Consequently, p72 expression led to an increase in MAPK signaling and cell proliferation whereas p66 expression resulted in reduced MAPK activation and decreased cell proliferation. In other words, the p66 isoform of NUMB is anti-proliferative and can potentially function as a tumor suppressor. In contrast, the p72 isoform may assist in sustaining ALK activity and thereby contributing to tumorigenesis.

**Figure 8:**
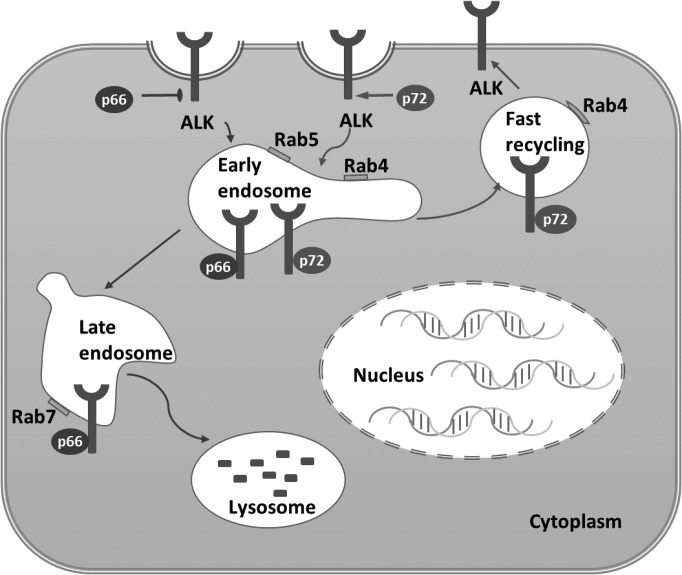
A model of NUMB-regulated ALK endocytosis and recycling.

It is intriguing that p66-NUMB and p72-NUMB exhibit distinct roles on ALK endosomal recycling and lysosomal degradation. The two isoforms differ in the absence or presence of a 48aa insert (coded by exon 9) in the PRR (Verdi et al., 1999). Based on our data, we hypothesize that p72-NUMB and p66-NUMB may recruit different endosomal sorting factors following ALK internalization. In this regard, it should be noted that the two isoforms do not necessarily play opposing roles in the ALK endosomal sorting, but more likely, they prefer, to a significant degree, the recycling or late endosomal compartment respectively. A mass spectrometry-based approach has been employed to quantitatively compare the interactome between p72-NUMB and p66-NUMB. Indeed, while both isoforms were found capable of binding to several known NUMB-interacting endocytic proteins, the p72-NUMB bound significantly more strongly to EPS15 and AP-2 (Krieger et al., 2013). These data support the possibility that NUMB isoforms may act differently during endocytosis and reveal that certain additional effectors that may be involved in these functional differentiations. It is therefore likely that the presence of the PPR insert skews the p72-NUMB towards binding to factors, which allows it to be sorted to the recycling endosome, whereas the absence of the insert results in the sorting of p66-NUMB to the late endosome and ultimately to the lysosome for degradation (Figure 8).

It was recently reported that alternative NUMB exon 9 splicing led to differential expression of the NUMB-PRR^S^ (p66) and NUMB-PRR^L^ (p72) isoforms with opposite effects on the growth and colony formation of breast and lung cancer cells (Bechara et al., 2013). While the latter (p72) was shown to promote the proliferation and colony formation by MCF7 and A549 cells, the former (p66) inhibited growth and decreased the colonogenic capacity of these cells (Bechara et al., 2013). It was suggested that this NUMB isoform-dependent effect on tumorigenicity might be mediated by NOTCH (McGill et al., 2009). Our work provides further support to this notion as the two NUMB isoforms may have distinct roles in the endocytosis, degradation, and recycling of NOTCH. Indeed, the mechanism identified herein for ALK may be used by NUMB to regulate the trafficking and activity of a large number of receptor tyrosine kinases (RTKs) that NUMB can potentially bind (Wei et al., 2018). A model is therefore emerging whereby the PTB domain of NUMB mediates partner binding whereas the PRR region controls the trafficking of the bound protein in an isoform-dependent manner (Colaluca et al., 2018). Given that RTKs constitute a major portion of the NUMB-PTB interactome (Wei et al., 2018) and are involved in the pathogenesis of numerous types of cancer, the NUMB isoform-specific pro- or anti-tumorigenesis role (Westhoff et al., 2009; Misquitta-Ali et al., 2011; Bechara et al., 2013) may be exploited for prognostic or therapeutic applications. In this regard, it would be interesting to find out if a higher ratio of expression of p66/p65-NUMB over p72/p71-NUMB could predict better outcome for the corresponding cancer. It is also conceivable that blocking the interaction of p72/p71-NUMB with the factors that mediate early endosome sorting would present an attractive therapeutic strategy by rewiring the endocytic pathway for RTK to degradation in the lysosome.

## Materials and methods

### Cell surface protein biotinylation and streptavidin pulldown

Cell surface ALK expression level was examined using the EZ-Link NHS-SS-biotin. The cells grown to 70% confluency were put at 4°C for 30 min, washed with cold PBS buffer, and then labeled with EZ-Link NHS-SS-biotin (Thermo Scientific, catalogue number: 21331) in biotinylation buffer (154 mM NaCl, 10 mM HEPES, 3 mM KCl, 1 mM MgCl_2_, 0.1 mM CaCl_2_, 10 mM glucose, pH 7.6) for 1 h at 4°C. After washing with cold PBS buffer, the free biotin was quenched by biotin stopping buffer (10% FBS and 100 mM Glycine in DMEM) for 5 min on ice. After washing with cold PBS buffer, the cells were lysed and incubated with streptavidin resin for 2 h to pull down the biotinylated proteins. The streptavidin beads were collected by centrifugation (3000* g*) and washed three times with cell lysis buffer. The isolated biotinylated proteins were detached from streptavidin beads by boiling in 2× SDS-Laemmli sample buffer, separated by SDS-PAGE and analyzed by western blot.

### Biotinylation internalization assay

Cells were labeled with biotin as mentioned above and incubated at 18°C in DMEM containing 10% FBS and 25 mM HEPES, pH 7.6 for different period of times to allow biotinylated surface protein to accumulate in early or sorting endosomes (McGill et al., 2009). At the indicated times, the medium was removed and the cells were put on ice and washed with cold PBS buffer. The remaining cell surface biotinylation was stripped by two 20-min washings in biotin stripping buffer (60 mM Glutathione, 100 mM NaCl, 75 mM NaOH, 10% FBS, 1 mM MgCl_2_, 0.1 mM CaCl_2_) at 4°C. Streptavidin pulldown and western blot were performed as described above.

### Recycling assay

The biotinylated cells were incubated at 18°C for 2 h in DMEM containing 10% FBS and 25 mM HEPES, pH 7.6. To know the amount of internalized biotinylated protein, one dish of cells was incubated in biotin stripping buffer for 20 min twice to strip biotin from biotin labeled cell surface proteins and lysated in cell lysis buffer. Another two dishes of cells were put in cell culture incubator for 30 min. One dish of cells was lysed in lysis buffer directly. The other dish of cells was first stripped in stripping buffer for 20 min twice and then lysed in lysis buffer. Then streptavidin pulldown and western blot assays were performed.

### Soft-agar assay for colony formation

First, 1% agar and 0.7% agar were prepared and autoclaved. For the base layer, 1% agar was melted and cooled to 40°C in a water bath. Meanwhile, 2× RPMI with 20% FBS and 200 μg/ml penicillin/streptomycin were warmed in a 40°C water bath. Next, equal volumes of the two solutions were mixed to result in a 0.5% agar, 1× RPMI with 10% FBS mixture, and 1.5 ml of the mixture was quickly added to each well of a 6-well plate. For the top layer, 0.7% agar was used for making the mixture. Cells were trypsinized and the cell density was adjusted to 200000 cells/ml in warm PBS buffer. Then 30000 cells were suspended gently in 9 ml of a mixture containing 0.35% agar, 1× RPMI, and 10% FBS, and 1.5 ml of the mixture was quickly added to each well of 6-well plate on top of the base layer. The plates were then incubated at 37°C in a humidified atmosphere containing 5% carbon dioxide and fed with 1× RPMI medium containing 10% FBS, 100 μg/ml penicillin/streptomycin, and L-glutamine. After 10–30 days, when the colonies grew to proper sizes, the plates were stained with 0.005% crystal violet for 1 h before microscopy.

### Statistics

All the statistical tests were done using Student’s *t*-test.

## Supplementary Material

mjz003_Supplementary_material
