# Supplementary material for: NUMB regulates the endocytosis and activity of the anaplastic lymphoma kinase in an isoform-specific manner
Source: J Mol Cell Biol. 2019 Feb 6;11(11):994–1005. doi: 10.1093/jmcb/mjz003 (PMC6927325; doi:10.1093/jmcb/mjz003)
Supplement: mjz003_Supplementary_material [file mjz003_supplementary_material.pdf]

NUMB regulates the endocytosis and activity of the anaplastic lymphoma kinase in an isoform-specific manner

Ran Wei, Xuguang Liu, Courtney Voss, Wentao Qin, Lina Dagnino, Lei Li, Marc Vigny, and Shawn Shun-Cheng Li

SUPPLEMENTARY INFORMATION

This file contains Supplementary materials and methods.

Antibodies

The antibodies used in this study include rabbit anti-NUMB (H-70, Santa Cruz, catalogue number: sc-25668), mouse anti-HA (Santa Cruz, catalogue number: sc-7392), rabbit anti-β tubulin (Santa Cruz, catalogue number: sc-9104), mouse anti-Erk (Cell signalling, catalogue number: 4696), mouse anti-pErk (Cell signaling, catalogue number: 9106), mouse anti-Flag (Sigma, catalogue number: F7425), mouse anti-GFP (B-2, Santa Cruz, catalogue number: sc-9996), and mouse anti-ALK (mAb46, generated by the Vigny lab).

Cell culture

IMR-5 cells were grown in Dulbecco's Modified Eagle Medium (DMEM) supplemented with 10% fetal bovine serum (FBS, Wisent Bioproducts, catalogue number: 098150), 100 μg/ml penicillin/streptomycin and L-glutamine (2 mM). HEK293 cells were grown in Roswell Park Memorial Institute (RPMI) 1640 medium supplemented with 10% FBS, 100 μg/ml penicillin/streptomycin and L-glutamine (2 mM). Cells were incubated at 37 ℃ in a humidified atmosphere containing 5% carbon dioxide.

Transient transfection

The plasmid DNA was first diluted in serum-free Minimum Essential Media (MEM) to a final concentration of 0.01 μg/ μl. For each 10-cm dish, 40 μl of X-tremeGENE HP DNA transfection reagent (Roche) was mixed with 1ml plasmid DNA in MEM. The mixture was added to the culture dish (10ml) after 15 min of incubation at room temperature. Following transfection, cells were incubated for 48~72 hours to allow the ectopic gene expression.

siRNA interference

siRNA (5'-GGACCUCAUAGUUGACCAG-3') (obtained from sigma) was synthesized to target several Numb isoforms including p66-NUMB and p72-NUMB. For each 35-mm dish, siRNA was first diluted with Opti-MEM I (Gibco) to a final concentration of 2.5 μM in a 200 μl total

volume. 5 μl DharmaFECT reagent (GE) was added to 195 μl serum-free MEM and gently mixed. The siRNA and reagent were then mixed and added to the culture dish (1.6 ml) after 15 min incubation at room temperature. Cells were incubated for 48~72 hours before further analysis.

Immunoprecipitation and Western blotting

The cultured cells were lysed in cold mammalian cell lysis buffer (1% Triton X-100, 50 mM Tris-pH 7.2, 150 mM NaCl, 2 mM $MgCl_2$, 0.1 mM EDTA, 0.1 mM EGTA, 0.5 mM DTT, 10 mM NaF) containing complete protease inhibitors (Roche). Cell debris was removed by centrifugation, and 500 μg of supernatant protein was incubated for four hours in the presence of 1 μg antibody and 30 μl of 50% slurry protein G-Sepharose beads (Roche) at 4 ℃. The beads were subsequently washed three times with lysis buffer and boiled with SDS-loading dye. The immunoprecipitates were resolved by SDS-PAGE. All proteins were then transferred to a PVDF membrane by semi-dry transferring method (Bio-Rad), detected by immunoblotting with appropriate antibodies, and visualized by Enhanced Chemiluminescence (ECL).

Bacterial expression of recombinant proteins and GST pull-down

The BL21 strain of *Escherichia coli* was transformed with pGEX6P3-NUMB (PTBi/PTBo). Positive colonies were cultured in Lysogeny Broth (LB) medium at 37 ℃ to reach OD_{600} 0.6~0.8, then the cultures were induced with 0.5 mM IPTG for 16 hours at 18 ℃. The bacterial cells were harvested and pellets were suspended in PBS buffer containing complete protease inhibitors (Roche). Triton X-100 was added to a final concentration of 2%, lysozyme was added to a final concentration of 1 mg/ml and benzonase was added to a final concentration of 20 units/ml. The suspension was sonicated six times (10 seconds each) on ice and then lysed for 30 min at room temperature. The lysates were centrifuged at 15,000 × g for 30 min at 4 ℃ and supernatant was collected. Purification of GST-tagged proteins was performed with glutathione resin (GE healthcare). The resin was washed with PBS buffer three times. Lysate was then loaded to the resin, followed by three washes with PBS buffer. To determine the purity of GST-proteins, a small amount of resin was boiled with SDS-loading dye and then analyzed by SDS-

PAGE/Coomassie staining. Pull-down was performed by incubating mammalian whole cell lysate with GST-NUMB PTBi, GST-NUMB PTBo or GST beads for 4 hours at 4 ℃. This was followed by three washes with mammalian cell lysis buffer. Proteins bound to GST or GST-PTB were resolved on SDS-PAGE and identified via Western Blotting.

Gene truncation and site-directed mutagenesis

Truncated forms of NUMB were constructed by standard PCR and re-ligation procedures. Five gene mutants – p66-NUMB-F162V, p72-NUMB-F162V, ALK-N1477A, ALK-N1583A, ALK-1477A/1583A – were constructed by whole plasmid site-directed mutagenesis. Numb F162V mutation primers are: 5'-CCGTGGGCTGTGCTGTTGCAGCCTGTTTAG-3' and 5'-CTAAACAGGCTGCAACAGCACAGCCCACGG-3'. ALK-N1477A mutation primers are 5'-GGAGGGGGACACGTGGCCATGGCATTCTCTCAGTC-3' and 5'-GACTGAGAGAATGCCATGGCCACGTGTCCCCCTCC-3'. ALK-N1583A mutation primers are 5'-CTTCCCTTGTGGGAATGTCGCCTACGGCTACCAGCAACAG-3' and 5'-CTGTTGCTGGTAGCCGTAGGCGACATTCCCACAAGGGAAG-3'. A pair of complementary mutagenic primers were designed for individual mutants. The entire plasmids were amplified using high fidelity DNA polymerase. Next, Dpn1 enzyme was used to digest the methylated template plasmid. Undigested plasmids were recycled and used for transformation. Positive colonies were identified by gene sequencing.

Immunostaining and confocal microscopy

Cells were cultured in 35-mm glass-bottomed dishes (P35G-1.0C, Matek) under standard conditions. When cells grew to the 20%~40% confluence, they were fixed with 4% paraformaldehyde and permeabilized with 0.2% triton X-100 for 10~30 mins. The cells were rinsed with PBS buffer three times and incubated with 3% BSA in PBS to block non-specific signals. The primary antibodies were diluted with 3% BSA in PBS at 1:50~ 1:200 ratios and added to the dishes for overnight incubation. The cells were rinsed with PBS buffer three times and incubated with secondary fluorescent antibodies (Invitrogen, 1:1000 dilution) for 1 hour. The cells were rinsed with PBS buffer three times and covered with mounting medium containing

DAPI (4',6-diamidino-2-phenylindole) fluorescent dye. For microscopic observation, a Zeiss Meta 510 LSM was used. To standardize images captured, all fluorescence images from different samples were obtained under the same microscope setting. Z-stack scanning was applied to all samples with the same step distance.

All images were processed and analyzed by ImageJ software (imagej.nih.gov/ij/). Co-localization images were merged from the same z-stack slices in two different channels. For co-localization quantification, the Pearson product-moment correlation (Pearson's) was quantified by the JACoP (Just Another Colocalization Plugin) plugin of ImageJ software. From the unprocessed images, individual cells were picked by the ROI (Regions of Interest) manager plugin and their Pearson's coefficient values were determined by the JACop plugin under standard settings.

Peptide synthesis

Both free and membrane-bound peptides were synthesized using an automatic Intavis AG workstation. All individual amino acids were in Fmoc (9-fluorenylmethyloxycarbonyl)-protecting forms. For free peptide synthesis, Rink-resin (Rink-NH_2) was used to couple the first amino acid, whereas for on-membrane peptide synthesis, the amine-derivate cellulose membrane (cellulose-NH_2) was made to couple the first amino acid. Generally, in each synthesis cycle, the carboxyl group of Fmoc-protecting amino acid (Fmoc-R-COOH) was first linked to the amine group of the previous amino acid (or Rink-NH_2) through an amide bond. All un-occupied amine groups were then blocked (acetylated) by acetic anhydride to prevent any incorrect amide bonds forming in subsequent cycles. Next, the Fmoc group was removed by piperidine (de-protecting) to release the free amide group which would be ready for linking the carboxyl group of the next amino acid. Fluorescein-NHS was linked to the amine group of the last amino acid in free peptide synthesis. After synthesis, the on-membrane peptides were treated with a mixture containing 47.5% TFA (trifluoroacetic acid), 1.5% TIPS (tri-isopropylsilane) and 51% water, which would remove all other protecting groups on amino acid side chains. The free peptides were treated with a mixture containing 95% TFA, 3% TIPS and 2% water to free side chains and cut off the Rink-resin.

Dissociation constant determination

The dissociation constant between protein and peptide was determined by fluorescence polarization (FP) assay. The proteins and fluorescein labelled peptides were prepared following the procedures introduced above. To reduce variation, two independent tests were prepared simultaneously. 384-well flat bottom plates (Corning-3575) were used for sample reading and a PerkinElmer Envision 2103 plate reader was used for evaluating FP. The peptides (approximately 2 μM each) were dissolved in 100 μl DMSO and 3 μl of this was diluted 100-fold in water. The concentrations of the proteins were determined and adjusted to 50 μM. Two-fold serial dilutions were set up to create 16 protein concentrations ranging from 0 to 50 μM. 30 μl of each protein sample was mixed with 5 μl peptide in the 384-well plate and the FP was determined by the plate reader. We used an approximate equation for non-linear fitting by assuming that the concentration of peptide was much lower than the K_D value:

$$\Delta FP = FP_{obs} - FP_0 = FP_{max} \times [\text{domain}] / (K_D + [\text{domain}])$$

The full equation without the approximation can be found in this study (Kaushansky et al., 2010).

Cell proliferation assay

Overall cell viability was evaluated using Sigma WST-8 ((2-(2-methoxy-4-nitrophenyl)-3-(4-nitrophenyl)-5-(2,4-disulfophenyl)-2H-tetrazolium, monosodium salt), which produced a water-soluble formazan dye in the presence of cellular dehydrogenase. Cells were cultured in 100 μl medium within 96-well plates. 10 μl of WST-8 solution was directly added to the 100 μl medium; cells were then returned to the incubation chamber for another 30 min to 1 hour. The 96-well plates were then read for absorbance at 460 nm, which was proportional to the total number of viable cells.
